# Pathways to Suicide-Related Behavior in Offspring of Mothers With Depression: The Role of Offspring Psychopathology

**DOI:** 10.1016/j.jaac.2015.02.006

**Published:** 2015-05

**Authors:** Gemma Hammerton, Stanley Zammit, Liam Mahedy, Rebecca M. Pearson, Ruth Sellers, Anita Thapar, Stephan Collishaw

**Affiliations:** a Institute of Psychological Medicine and Clinical Neurosciences, Cardiff University, Cardiff, UK; b Centre for Academic Mental Health, University of Bristol, Bristol, UK

**Keywords:** suicide-related behavior, ALSPAC, maternal depression, psychopathology

## Abstract

**Objective:**

Offspring of mothers with depression are a high-risk group for the development of suicide-related behavior. These offspring are therefore a priority for preventive interventions; however, pathways contributing to risk, including specific aspects of offspring psychopathology, remain unclear. The aim of this study was to examine whether offspring symptoms of major depressive disorder (MDD), generalized anxiety disorder (GAD), disruptive behavior disorder (DBD), attention-deficit/hyperactivity disorder (ADHD), and alcohol abuse independently mediate the association between maternal depression and offspring suicide-related behavior.

**Method:**

Data were used from a population-based birth cohort, the Avon Longitudinal Study of Parents and Children (ALSPAC). Three distinct classes of depression symptoms across the mothers’ first 11 years of their child’s life were identified (minimal, moderate, chronic-severe). Offspring psychopathology was assessed at age 15 years and suicide-related behavior at age 16 years. Data were analyzed using structural equation modeling.

**Results:**

There was evidence for increased risk of suicidal ideation in offspring of mothers with chronic-severe depression symptoms in comparison to offspring of mothers with minimal symptoms (odds ratio = 3.04, 95% CI = 2.19, 4.21). This association was independently mediated by offspring MDD, GAD, and DBD symptoms. The same mechanisms were found for offspring of mothers with moderate depression symptoms over time. Results were similar for offspring suicide attempt except for additional evidence of an indirect effect through offspring ADHD symptoms.

**Conclusion:**

Findings highlight that suicide prevention efforts in offspring of mothers with depression should not only be targeted at offspring with MDD; it is also important to consider offspring with other forms of psychopathology.

It is well established that offspring of mothers with depression are at increased risk for the development of suicide-related behavior^1^ and are therefore a priority for preventive interventions. However, the reasons why they are at increased risk, including the role of specific aspects of offspring psychopathology, remain unclear. Offspring of mothers with depression are at increased risk for a range of mental health problems (including depression, anxiety, disruptive behavior disorders, attention-deficit/hyperactivity disorder (ADHD), and alcohol abuse),^2,3^ and there is some evidence that each of these disorders is associated with both suicidal ideation and suicide attempt in adolescence.^4-7^ Studies have consistently shown that depressive disorder is associated with suicide-related behavior when taking account of other psychopathology^5,6^; however, findings as to whether the effects of other disorders are independent of depression and each other have been inconsistent. A recent national survey of US adolescents^6^ found no independent effect of generalized anxiety disorder (GAD), assessed retrospectively, on suicide-related behavior in adolescents. However, a longitudinal sample of adolescents^7^ found that GAD was associated with later suicide-related behavior after adjusting for the presence of other psychopathology. Findings for substance abuse have also been inconsistent, with some studies showing an association with suicidal ideation^6^ and others only with suicide attempt.^4,5^ ADHD has generally been combined with other disruptive behavior disorders,^4,5^ meaning that it is difficult to draw conclusions about the independent effects; however, the national survey of US adolescents^6^ examined the disorders separately and found an independent effect of oppositional defiant disorder (ODD) on later suicidal ideation but that ADHD was associated only with the transition from ideation to attempt.^6^ Previous studies, however, have not fully taken account of the co-occurrence of offspring psychopathology by examining the effects of each disorder while adjusting for covariance between them. Given the evidence that disorders tend to co-occur in adolescents^8^ and that high levels of comorbidity are associated with suicide,^9^ taking account of this co-occurrence is important to examine the independent effects of specific aspects of offspring psychopathology.

Longitudinal studies examining the influence of maternal depression on offspring suicidal ideation have found that adjustment for offspring depression symptoms did not fully account for risk effects^10,11^; however, the mediating role of other types of offspring psychopathology has rarely been investigated. One longitudinal case-control study using Swedish inpatient care registers found that the association between maternal affective disorder and offspring suicide attempt was attenuated, although still present, when adjusted for whether offspring had been hospitalized because of psychiatric disorder.^12^ A cross-sectional study found a similar pattern of results when examining suicidal ideation and suicide attempt in adult offspring of parents with depression, with adjustment for the presence of lifetime offspring psychiatric disorder.^13^ However, more research is needed to examine the relative or differential importance of specific types of offspring psychopathology in explaining the association between maternal depression and subsequent offspring suicidal ideation and suicide attempt in adolescence. This is important, given that offspring of mothers with depression show a broad range of psychopathology. In addition, it is an essential first step to establishing whether treating specific symptoms in offspring of mothers with depression is likely to lead to a reduction in suicide-related behavior. There is evidence that more chronic and severe symptoms of maternal depression have a greater impact on offspring development,^14^ highlighting the importance of testing mechanisms for this group. However, it is not known whether risk mechanisms’ underlying links with offspring suicide-related behavior vary by maternal depression severity.

Therefore, the present investigation uses a large population cohort to examine how much of the association between differing levels of maternal depression symptoms, over the first 11 years of their child’s life, and later offspring suicidal ideation and suicide attempt is explained by proximal offspring psychopathology, including symptoms of major depressive disorder (MDD), GAD, disruptive behavior disorder (DBD), ADHD, and alcohol abuse.

## Method

### Study Sample

Data were used from a large UK birth cohort study: the Avon Longitudinal Study of Parents and Children (ALSPAC). The cohort was set up to examine genetic and environmental determinants of health and development.^15^ The core enrolled sample consisted of 14,541 pregnant women resident in the former county of Avon, United Kingdom, who had an expected date of delivery between April 1, 1991, and December 31, 1992. Of the 14,062 live births, 13,617 were singletons and were alive at 1 year of age. The sample is broadly representative of the UK population; however, mothers enrolled in ALSPAC were more likely to live in owner-occupied accommodation and to have a car, more likely to be married, and more likely to be white.^16^ Parents and children have been followed up regularly since recruitment via questionnaire and clinic assessments. All adult participants gave informed consent, and ethical approval for the study was obtained from the ALSPAC Ethics and Law Committee and the local research ethics committees. Further details on the sample characteristics and methodology have been described previously,^15,16^ and detailed information about ALSPAC can be found on the study website (http://www.bristol.ac.uk/alspac). Information on all available ALSPAC data is available on the fully searchable data dictionary (http://www.bris.ac.uk/alspac/researchers/data-access/data-dictionary).

### Measures

#### Maternal Depression Symptom Trajectories

Maternal depression symptoms were assessed at 10 time points (from 18 weeks’ gestation to child age 11 years) using the Edinburgh Postnatal Depression Scale (EPDS).^17^ In a prior analysis using this dataset, latent class growth analysis was used to identify qualitatively distinct patterns of depression symptoms in mothers over time. Further details on the derivation and validation of classes are given in Supplement 1 and Table S1, available online. For the purposes of these analyses, 3 trajectory classes of depression are compared: mothers with chronic and severe levels of symptoms (“chronic-severe”), those with subthreshold but sustained depression symptoms over time (“moderate”), and those with very low levels of depression symptoms (“minimal”). Approximately 40% of the sample belonged to the minimal class, 55% of the sample belonged to the moderate class, and 5% of the sample belonged to the chronic-severe class. In all analyses, the minimal class is treated as the reference group. Table S2 (available online) shows mean depression symptoms at each assessment for mothers in each of the groups.

#### Offspring Suicide-Related Behavior

Suicide-related behavior at age 16 years was assessed via a self-report postal questionnaire.^18^ Participants were classified as having a lifetime history of suicidal ideation if they responded positively to either of the following questions: Have you ever found yourself wishing you were dead and away from it all? Have you ever thought of killing yourself, even if you would not really do it? The present investigation focuses on children who reported suicidal ideation in the previous year only (78% of those who reported lifetime suicidal ideation by age 16 years) to preserve the time ordering of the analysis. History of suicidal ideation at age 11 years was assessed using the childhood interview for borderline personality disorder with the question, Have you thought about killing yourself?

Secondary analyses investigated specific associations with lifetime history of suicide attempt by age 16 years. Participants were classified as having a lifetime history of a suicide attempt if they endorsed the following question: On any of the occasions when you have hurt yourself on purpose, have you ever seriously wanted to kill yourself? Participants were also included if they reported “I wanted to die” as a reason to explain why they hurt themselves on purpose on the most recent occasion.

#### Possible Mediating Variables: Offspring Psychopathology

Offspring psychopathology was assessed using the Development and Well-Being Assessment (DAWBA)^19^ parent and child versions. The DAWBA is a semi-structured interview consisting of questions about child mental health symptoms and their impact. Different time spans are used across the DAWBA sections to follow *DSM-IV* criteria. At age 15 years, parent versions of the DAWBA were used to assess symptoms of DBD (ODD over the past 6 months or conduct disorder over the past year) and symptoms of ADHD over the past 6 months. Child versions of the DAWBA were used to assess symptoms of MDD over the past month and symptoms of GAD over the past 6 months. Each DAWBA section consisted of 20 to 25 questions that followed the diagnostic criteria operationalized in the *DSM-IV* or *ICD-10*. Continuous symptom scores were derived from the sum of all symptom items within the relevant section of the DAWBA. Symptoms of alcohol abuse over the last 2 years were assessed at the same time point using questions taken from the Semi-Structured Assessment of the Genetics of Alcoholism interview that correspond relatively well to *DSM-IV* criteria for alcohol abuse and dependence.

#### Potential Confounders

Potential socio-demographic and familial confounding factors assessed in pregnancy were chosen based on evidence from previous literature.^20,21^ Maternal questionnaires completed during pregnancy were used to assess housing tenure (owned versus rented), marital status (married versus single), maternal level of education (below O-level, O-level, or above O-level, where O-level denotes completion of secondary schooling), self-reported psychiatric disorder before pregnancy (yes/no, including drug addiction, alcoholism, schizophrenia, anorexia nervosa, severe depression, or any other psychiatric disorder), maternal family history of depression (neither, one, or both parents), and smoking in pregnancy (smoked tobacco in either the first 3 months or the last 2 weeks of pregnancy).

### Data Analysis

The starting sample for these analyses included mothers who had information on the latent classes of maternal depression symptoms (N = 10,559). Of the starting sample, 4,588 offspring had complete data on suicide-related behavior at age 16 years (43%; 1,904 males and 2,684 females; mean age = 16.7 years, SD = 0.2 years). Of these individuals, 2,445 offspring also had complete data on symptoms of psychopathology at age 15 years (Figure S1, available online). Given that listwise deletion of families can increase sample bias,^22^ missing data for offspring suicide-related behavior and psychopathology and other covariates were imputed using multivariate imputation by chained equations,^23^ which assumes that data are missing at random (MAR; i.e., given the observed data included in the imputation model, the missingness mechanism does not depend on the unobserved data^22^). Missing data were found to be dependent on several observed variables available for the full cohort at birth (listed in Supplement 2, available online); therefore, these were included in the imputation model to make the assumption of MAR as plausible as possible. In addition, the imputation model included all variables included in the analysis models, and a number of auxiliary variables that were associated with offspring suicide-related behavior and psychopathology.^22^ Further detail on the imputation procedure is given in Supplement 2 and Table S3, available online. Main results are presented for 4 different imputation approaches (full imputation, N = 10,559; imputation for those individuals sent questionnaires at age 16, n = 8,475; imputation for those with complete outcome data, n = 4,588; and complete case analysis, n = 2,445).

Following multiple imputation, symptom counts were standardized to allow estimates to be comparable. Univariable logistic and linear regression analyses were then performed, as appropriate, to examine initial associations between variables. Next, a multiple mediation model was run using structural equation modeling (SEM) in M*plus* to assess the effects of moderate or chronic-severe maternal depression symptoms (with minimal class as the reference group) on offspring past-year suicidal ideation at age 16 years. Models include both direct and indirect paths through offspring symptoms of psychopathology at age 15 years and simultaneously adjust for residual covariance between symptoms. A weighted least-squares estimator was used because of its robustness in analyzing both continuous and categorical measures in SEM.^24^ Results from path analyses with a categorical outcome (including indirect effects) are presented as probit regression coefficients. Indirect effects were calculated using bias-corrected bootstrapping with 500 replications. To examine whether the indirect effects within the same model differed in strength, post-hoc Wald χ^2^ tests were used to test the assumption of equality between constrained indirect effects. Secondary analyses examined offspring lifetime suicide attempt as the outcome. Analyses were conducted using Stata version 13^25^ and M*plus* version 7.^24^

## Results

### Offspring Suicidal Ideation and Psychopathology

The number of adolescents who reported past-year suicidal ideation at the age 16 years assessment was 672 of 4,588 (15%; 174 males and 498 females), and 272 of 5,613 children (5%; 144 males and 128 females) reported lifetime suicidal ideation at age 11 years. The overall prevalence was very similar in fully imputed models taking account of missing data; the estimated prevalence for past-year suicidal ideation at age 16 years was 15% (95% CI = 14%−17%; 11% of males and 20% of females), and the estimated prevalence for lifetime suicidal ideation by age 11 years was 6% (95% CI = 5%−6%; 6% of males and 5% of females). Table S4 (available online) shows means, SDs, and correlations between offspring symptoms of psychopathology at age 15 years.

#### Associations Between Maternal Depression Classes, Offspring Psychopathology, and Offspring Suicidal Ideation

In all, 29% (95% CI = 23%–35%) of offspring of mothers with chronic-severe depression symptoms reported past-year suicidal ideation at age 16 years, compared to 17% (95% CI = 15%−18%) of offspring of mothers with moderate depression symptoms and 12% (95% CI = 10%−13%) of mothers with minimal symptoms over time. There was evidence for increased risk of suicidal ideation in offspring of mothers with chronic-severe depression symptoms (odds ratio [OR] = 3.04, 95% CI = 2.19–4.21) and offspring of mothers with moderate symptoms (OR = 1.51, 95% CI = 1.30–1.75) in comparison to offspring of mothers with minimal symptoms (Table 1). Table 1 also shows evidence of a positive association between offspring symptoms of MDD, GAD, DBD, ADHD, and alcohol abuse at age 15 years and offspring suicidal ideation at age 16 years.

Table S5 (available online) shows evidence for increased risk of all symptoms of psychopathology at age 15 years in offspring of mothers with chronic-severe depression symptoms and offspring of mothers with moderate symptoms in comparison to offspring of mothers with minimal symptoms.

#### Mediation of Effect of Maternal Depression Classes on Offspring Suicidal Ideation

**Figure 1:**
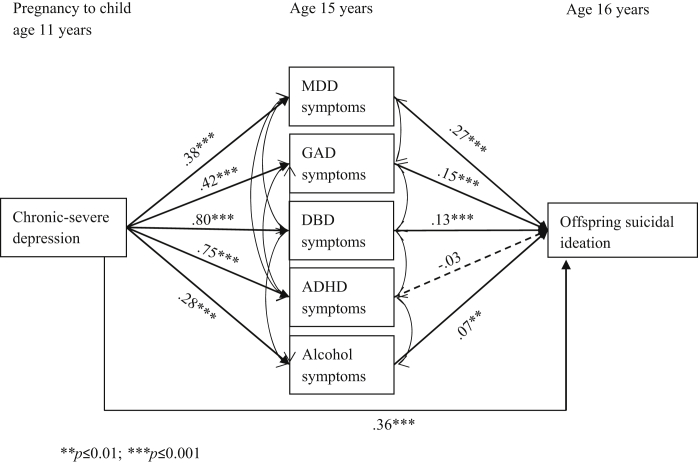
Structural model showing the direct effect of maternal chronic-severe depression symptoms (with minimal class as the reference group) on offspring past-year suicidal ideation, and the indirect effects through offspring symptoms of major depressive disorder (MDD), generalized anxiety disorder (GAD), disruptive behavior disorder (DBD), attention-deficit/hyperactivity disorder (ADHD), and alcohol abuse. Note: Residual covariance coefficients not shown on diagram; imputed N = 10,559.

Next, a multiple mediation model was run to assess the effects of the latent classes of maternal depression symptoms (with minimal class as the reference group) on subsequent offspring past-year suicidal ideation (at age 16 years) both directly and indirectly through offspring symptoms of psychopathology at age 15 years. Figure 1 shows results from the structural model examining the direct effect of maternal chronic-severe depression symptoms on offspring suicidal ideation, and the indirect effects through offspring symptoms of MDD, GAD, DBD, ADHD, and alcohol abuse. There was evidence that offspring of mothers with chronic-severe symptoms were at increased risk for all symptom types compared to offspring of mothers with minimal symptoms. Figure 1 also shows evidence that offspring symptoms of MDD, GAD, DBD, and alcohol abuse were independently associated with offspring suicidal ideation. However, there was still evidence of a direct effect of maternal chronic-severe depression on offspring suicidal ideation not mediated through offspring symptoms of psychopathology (probit coefficient [B] = 0.36; 95% CI = 0.17–0.55, *p* < .001).

Table 2 (model 1a) shows the unadjusted indirect effects of maternal chronic-severe depression on offspring suicidal ideation through offspring symptoms of MDD, GAD, DBD, ADHD, and alcohol abuse. There was evidence of an indirect effect through offspring symptoms of MDD (B = 0.10, 95% CI = 0.06–0.15), GAD (B = 0.06, 95% CI = 0.03–0.09), and DBD (B = 0.11, 95% CI = 0.06–0.16) and weak evidence of an indirect effect via symptoms of alcohol abuse (B = 0.02, 95% CI = 0.00–0.04). Table 2 (model 1b) shows that indirect effects via MDD, GAD, and DBD symptoms were slightly attenuated, but strong evidence of effects remained when adjusted for potential confounders assessed in pregnancy. Post-hoc Wald χ^2^ tests were then performed to compare the strength of parameters for the indirect effects with one another (again adjusting for potential confounders). There were no differences in effect sizes for the indirect effects via MDD, GAD, and DBD symptoms (all *p* ≥ .118); however, there was evidence that the indirect effects via MDD, GAD, and DBD symptoms were each stronger than the indirect effects through symptoms of ADHD and alcohol abuse (all *p* ≤ .037). Findings were comparable across different imputation samples (Table 2, models 2 and 3).

When using complete cases (Table 2, model 4), although all indirect effects were weaker, conclusions were similar. There was evidence again of an indirect effect through offspring symptoms of GAD (B = 0.04, 95% CI = 0.01–0.10) and DBD (B = 0.10, 95% CI = 0.05–0.18). However, there was no longer evidence of an indirect effect through offspring symptoms of MDD (B = 0.03, 95% CI = −0.03 to 0.08); this was due to a weaker association between maternal chronic-severe depression and offspring symptoms of MDD in complete case analyses (not shown).

The pattern of results was similar when examining indirect effects of maternal moderate depression symptoms on offspring suicidal ideation with evidence of an indirect effect through offspring symptoms of MDD (B = 0.05, 95% CI = 0.03–0.06), GAD (B = 0.03, 95% CI = 0.02–0.04), and DBD (B = 0.04, 95% CI = 0.02–0.06) after adjusting for confounders (Table S6, model 1b, available online). Findings were again comparable across different imputation samples (Table S6, models 2 and 3, available online) and when using complete cases (Table S6, model 4, available online). Post-hoc Wald χ^2^ tests showed evidence that the indirect effects via symptoms of MDD, GAD, and DBD were each stronger for offspring of mothers with chronic-severe depression symptoms compared to offspring of mothers with moderate symptoms (all *p* ≤ .034). Finally, when running all analyses only among those who reported no suicidal ideation by age 11 years, conclusions remained the same (available on request).

### Secondary Analyses

#### Mediation of Effect of Maternal Depression Classes on Offspring Lifetime Suicide Attempt

The number of adolescents who reported lifetime suicide attempt at the age 16 years assessment was 302 in 4,588 (7%; 61 males and 241 females). The overall prevalence was similar in fully imputed models taking account of missing data—the estimated prevalence for lifetime suicide attempt was 8% (95% CI = 7%−9%; 5% of males and 11% of females). Table 3 (model 1a) shows evidence of an indirect effect of maternal chronic-severe depression symptoms on offspring suicide attempt through offspring symptoms of MDD (B = 0.11, 95% CI = 0.06–0.16), GAD (B = 0.07, 95% CI = 0.03–0.11), DBD (B = 0.11, 95% CI = 0.06–0.16), and ADHD (B = 0.06, 95% CI = 0.01–0.12) and weak evidence of an indirect effect via symptoms of alcohol abuse (B = 0.02, 95% CI = −0.00 to 0.03). There was also evidence of a direct effect of maternal chronic-severe symptoms on suicide attempt not mediated through offspring symptoms of psychopathology (B = 0.31, 95% CI = 0.10–0.52, *p* = .003). Strong evidence of indirect effects via MDD, GAD, DBD, and ADHD persisted after adjustment for confounders (model 1b). Findings were comparable across different imputation samples (models 2 and 3).

When using complete cases (Table 3, model 4), all indirect effects were weaker. However, there was still evidence of an indirect effect through offspring symptoms of GAD (B = 0.04, 95% CI = 0.01–0.09) and DBD (B = 0.07, 95% CI = 0.02–0.15). There was only weak evidence of an indirect effect via ADHD symptoms (B = 0.03, 95% CI = −0.01–0.09), and again there was no longer evidence of an indirect effect through offspring symptoms of MDD (B = 0.03, 95% CI = −0.03 to 0.09) due to the weaker association between maternal chronic-severe depression and offspring symptoms of MDD in complete case analyses.

Finally, analyses with offspring suicide attempt were rerun only on the subsample of adolescents who had reported lifetime suicidal ideation by age 16 years. In all, 31% of adolescents who reported suicidal ideation in their lifetime also reported making a suicide attempt. Again, there was evidence of an indirect effect of maternal chronic-severe depression on offspring suicide attempt through offspring symptoms of ADHD (B = 0.17, 95% CI = 0.05–0.42) (full results available on request).

## Discussion

Children exposed to chronic and severe maternal depression symptoms across childhood are at considerably increased risk for suicidal ideation later in adolescence. This association was in part explained by offspring proximal symptoms of psychopathology. Importantly, however, there was still evidence of a direct effect of maternal chronic-severe depression not mediated by offspring proximal symptoms. A further novel and important finding was that closer examination of different aspects of psychiatric symptomatology showed that there were independent indirect effects through offspring symptoms not only of MDD but also of GAD and DBD after accounting for covariation of child symptomatology and potential confounding factors. In addition, the indirect effects through GAD and DBD symptoms were of equal importance to MDD symptoms in terms of explaining the link between maternal depression and adolescent suicidal ideation. This study found no evidence of an indirect effect on offspring suicidal ideation through offspring symptoms of ADHD or alcohol abuse over and above other symptoms in the offspring and potential confounders. Although effect sizes were weaker, mechanisms were similar for offspring of mothers with less severe levels of depression symptoms, with evidence of an indirect effect of maternal moderate depression symptoms on offspring suicidal ideation via offspring symptoms of MDD, GAD, and DBD after adjusting for potential confounders. Finally, findings were similar when investigating indirect effects on offspring suicide attempt in the whole sample, with the exception of an independent indirect effect of maternal depression on offspring suicide attempt via offspring symptoms of ADHD.

Consistent with previous literature,^12,13^ this study found evidence for a direct effect of maternal depression on offspring suicidal ideation after accounting for offspring psychopathology. However, this study extends prior research by examining the relative importance of specific aspects of offspring psychopathology in explaining the association between maternal depression and subsequent offspring suicidal ideation and suicide attempt in adolescence. The results extend studies that have examined whether the association is explained through offspring depression symptoms^10,11^ or psychiatric disorder^13^ and provides further evidence that mood, anxiety, and disruptive behavior disorders are independently associated with suicide-related behavior in adolescents after taking account of the co-occurrence of offspring symptoms. This study also adds to prior research by showing that the same mechanisms are important for offspring of mothers with subthreshold levels of depression symptoms over time. Contrary to our expectation that the strongest indirect effect would be through offspring MDD symptoms, there was no evidence that the indirect effect via MDD symptoms was stronger than via GAD or DBD symptoms. In the current study, offspring symptoms of ADHD mediated the association with offspring suicide attempt but not ideation. Given that studies often combine ADHD with ODD and conduct disorder,^4,5^ differential effects on suicidal ideation and suicide attempt may be missed. Our findings indicate that it may be more informative to examine ADHD separately from other disruptive behavior disorders when examining associations with suicide-related behavior. In addition, it was only ADHD symptoms that were associated with risk for suicide attempt among ideators. This supports previous findings that indicate ADHD is associated with the transition from ideation to attempt.^6^ It should be noted, however, that the sample size used in this study to examine risk for suicide attempt among ideators was small, given the complexity of the analyses. Therefore, findings are preliminary and require replication in larger samples. Previous findings regarding offspring alcohol abuse have been inconsistent. In this study, there was little evidence of an indirect effect through offspring symptoms of alcohol abuse for offspring suicidal ideation or attempt, mainly because of a lack of association between maternal depression and offspring symptoms of alcohol abuse after adjusting for confounders.

It was beyond the scope of this study to examine why maternal depression exerted an indirect effect on offspring suicide-related behavior through offspring symptoms of MDD, GAD, and DBD. Although mental health disorders may lead to suicidal ideation because of the distress or impairment that results from living with the disorder,^26^ this would not explain the weaker indirect effect found for symptoms of alcohol abuse or the differential effect of ADHD on offspring suicidal ideation and suicide attempt. Alternatively, it has been noted that the underlying mechanisms linking internalizing symptoms and externalizing symptoms to suicide-related behavior may differ.^27^ For example, hopelessness may explain the effects of internalizing symptoms on suicide-related behavior,^27^ whereas anger may be an important mechanism for the association between DBD symptoms and suicidal ideation.^28^ In addition, previous literature has hypothesized that the link between offspring ADHD and suicide attempt may be explained by the impulsivity and related deficits in executive functioning that are characteristic of ADHD.^29^ These factors would not necessarily index risk for suicidal thinking. Future research should investigate what might explain the indirect effects observed. In the current study, there was also evidence for a direct effect of maternal depression on offspring suicidal ideation and suicide attempt after accounting for proximal offspring psychopathology. This could be explained by the direct effect of exposure to maternal depression; however, this “direct” path could also be explained through other mediating pathways or by offspring psychopathology at other ages or measurement error in the assessment of symptoms at age 15 years.

The findings need to be considered in the light of several limitations. First, offspring symptoms of DBD and ADHD were parent reported, whereas MDD, GAD, and alcohol abuse were self-reported. Therefore, shared-rater bias may have affected associations between maternal depression and offspring symptoms of ADHD and DBD or between symptoms of MDD, GAD, and alcohol abuse and suicide-related behavior. Second, as with most cohort studies, there was selective attrition over time; however, potential bias arising from missing data was dealt with using multiple imputation, using a large amount of additional information to make the assumption of missing-at-random as plausible as possible.^22^ Findings from different imputation models and from complete case analyses were mostly comparable. However, it is important to note where differences were found. First, there was only weak evidence of an indirect effect on offspring suicide attempt via ADHD symptoms in complete case analyses. However, the indirect effect via ADHD symptoms was present in complete case analyses when examining risk for offspring suicide attempt within those who reported suicidal ideation. This finding therefore strengthens the conclusion regarding the importance of ADHD symptoms in predicting which offspring are most at risk for making a suicide attempt. Second, in complete case analyses, there was no evidence of an association between maternal chronic-severe depression and offspring depression symptoms. Previous studies using the same sample have reported that the association between maternal and offspring depression may be underestimated in complete case analyses because families in which both the mother and the child are depressed are more likely to have missing data.^30^ In addition, findings were comparable across each imputation sample, and under the MAR assumption, made more plausible by the variables used to predict missingness; multiple imputation should help correct for biases that may be present in complete case analyses.^31^ Third, in these analyses, although we adjusted for socio-demographic and familial measures, residual confounding by measurement error in these variables or by other unmeasured characteristics cannot be ruled out. Finally, although this study allowed for the time-ordering of effects to be examined, it is still important to consider the possibility of reverse causation (i.e., young people’s suicide-related behavior may have adverse effects on their own mental health and on maternal depression symptom course^32^). However, when excluding offspring who reported suicidal ideation by age 11 years, conclusions remained unchanged.

In this population-based sample, adolescent suicidal ideation and psychopathology were common in young people exposed to sustained periods of maternal depression in childhood. Clinicians treating mothers with ongoing depression symptoms should therefore consider the likelihood of psychopathology and potential risk of suicide-related behavior in offspring. Offspring proximal symptoms of MDD, GAD, and DBD all independently mediated the association between maternal chronic and severe depression symptoms and subsequent offspring suicide-related behavior. In addition, an indirect effect via ADHD symptoms was found for suicide attempt but not ideation. The same mechanisms were found to be important when mothers experienced less severe but sustained levels of depression symptoms over time. These results highlight that suicide prevention efforts in offspring of mothers with depression should be targeted not only at adolescents with depression symptoms but also those with other forms of psychopathology, such as anxiety or disruptive behavior symptoms. It is also important for clinicians to consider that offspring of mothers with depression may be at increased risk for making a suicide attempt if they have symptoms of ADHD. Knowledge about the unique contribution of different types of psychiatric symptoms also helps inform understanding of the mechanisms by which psychopathology might lead to suicide-related behavior in adolescents. Given that offspring proximal psychopathology did not completely explain the association between maternal depression and offspring suicide-related behavior in this study, future research should examine other mechanisms that may help to explain why offspring of mothers with depression are at increased suicide risk.

**Clinical Guidance**

• It is important to consider the likelihood of psychopathology and potential risk for suicide-related behavior in offspring of mothers with ongoing depression symptoms.
• Suicide prevention efforts in offspring of mothers with depression should be targeted not only at adolescents with depression symptoms but also those with other forms of psychopathology, such as anxiety or disruptive behavior symptoms.
• It is also important for clinicians to consider that offspring of mothers with depression may be at increased risk for making a suicide attempt if they have symptoms of ADHD.

## Figures and Tables

**Table 1:** Univariable Logistic Regression Analyses Between Each Class of Maternal Depression Symptoms in Comparison to Minimal Class (Reference Group) and Offspring Symptoms of Psychopathology (as the Exposures) and Offspring Past-Year Suicidal Ideation at Age 16 Years as the Outcome

Exposures	OR (95% CI)
Maternal depression class	
Minimal	Reference group
Moderate	1.51 (1.30, 1.75)∗∗∗
Chronic-severe	3.04 (2.19, 4.21)∗∗∗
Offspring psychopathology	
MDD symptoms	2.22 (1.99, 2.48)∗∗∗
GAD symptoms	1.83 (1.67, 1.99)∗∗∗
DBD symptoms	1.50 (1.38, 1.63)∗∗∗
ADHD symptoms	1.26 (1.14, 1.38)∗∗∗
Alcohol abuse symptoms	1.38 (1.27, 1.51)∗∗∗

Note: Imputed N = 10,559. ADHD = attention-deficit/hyperactivity disorder; DBD = disruptive behavior disorder; GAD = generalized anxiety disorder; MDD = major depressive disorder; OR = odds ratio.

∗∗∗ *p* ≤ .001.

**Table 2:** Indirect Effect of Maternal Chronic-Severe Depression (With Minimal Class as the Reference Group) on Offspring Suicidal Ideation Through Offspring Symptoms of Major Depressive Disorder (MDD), Generalized Anxiety Disorder (GAD), Disruptive Behavior Disorder (DBD), Attention-Deficit/Hyperactivity Disorder (ADHD), and Alcohol Abuse

Model^a^	Indirect Effects via Offspring Symptoms (Probit Coefficient [95% CI])				
	MDD	GAD	DBD	ADHD	Alcohol Abuse
Model 1a: using full imputed data; unadjusted (N = 10,559)	0.10 (0.06–0.15); *p* <.001	0.06 (0.03–0.09); *p* < .001	0.11 (0.06–0.16); *p* < .001	−0.02 (−0.06–0.02); *p* = .282	0.02 (0.00–0.04); *p* = .028
Model 1b: adjusted for confounders (N = 10,559)^b^	0.07 (0.04–0.11); *p* <.001	0.04 (0.01–0.07); *p* = .002	0.08 (0.04–0.12); *p* < .001	−0.01 (−0.04–0.03); *p* = .743	0.01 (−0.00–0.02); *p* = .158
Model 2: (n = 8,475)	0.10 (0.06–0.15); *p* <.001	0.06 (0.03–0.10); *p* < .001	0.10 (0.05–0.14); *p* < .001	−0.03 (−0.07–0.01); *p* = .200	0.02 (−0.00–0.03); *p* = .062
Model 3: (n = 4,588)	0.06 (0.00–0.11); *p* =.035	0.05 (0.01–0.08); *p* = .007	0.09 (0.04–0.14); *p* = .001	−0.02 (−0.06–0.01); *p* = .227	0.01 (−0.01–0.02); *p* = .355
Model 4: (n = 2,445)	0.03 (−0.03–0.08); *p* =.299	0.04 (0.01–0.10); *p* = .048	0.10 (0.05–0.18); *p* = .002	−0.05 (−0.12–−0.02); *p* = .031	0.00 (−0.01–0.02); *p* = .815

Note: Probit coefficients (B) and 95% CIs are displayed. Imputed N = 10,559.

a Model 1a shows the unadjusted results using the full imputed dataset; model 1b shows results after adjusting for confounders assessed in pregnancy; model 2 shows the unadjusted results using imputed data for those offspring who were sent the questionnaire at age 16 years; model 3 shows the unadjusted results using imputed data for mediators in those who had complete outcome data; model 4 shows the unadjusted results using only those with complete data on all variables in analysis.

b Adjusting for confounders assessed in pregnancy (child gender, housing tenure, marital status, maternal level of education, smoking in pregnancy, maternal family history of depression, and maternal psychiatric disorder before pregnancy).

**Table 3:** Indirect Effect of Maternal Chronic-Severe Depression (With Minimal Class as the Reference Group) on Offspring Suicide Attempt Through Offspring Symptoms of Major Depressive Disorder (MDD), Generalized Anxiety Disorder (GAD), Disruptive Behavior Disorder (DBD), Attention-Deficit/Hyperactivity Disorder (ADHD), and Alcohol Abuse

Model^a^	Indirect Effects via Offspring Symptoms (Probit Coefficient [95% CI])				
	MDD	GAD	DBD	ADHD	Alcohol Abuse
Model 1a: using full imputed data; unadjusted (N = 10,559)	0.11 (0.06–0.16); *p* < .001	0.07 (0.03–0.11); *p* < .001	0.11 (0.06–0.16); *p* < .001	0.06 (0.01–0.12); *p* = .022	0.02 (−0.00 to 0.03); *p* = .072
Model 1b: adjusted for confounders (N = 10,559)^b^	0.08 (0.04–0.12); *p* < .001	0.05 (0.02–0.08); *p* = .002	0.08 (0.04–0.13); *p* < .001	0.07 (0.02–0.12); *p* = .005	0.01 (−0.00 to 0.02); *p* = .288
Model 2: questionnaire (n = 8,475)	0.11 (0.06–0.16); *p* < .001	0.07 (0.03–0.10); *p* < .001	0.10 (0.05–0.15); *p* < .001	0.05 (0.01–0.10); *p* = .028	0.01 (−0.01 to 0.03); *p* = .190
Model 3: (n = 4,588)	0.06 (0.00–0.12); *p* = .037	0.06 (0.01–0.10); *p* = .007	0.08 (0.03–0.13); *p* = .002	0.04 (0.00–0.08); *p* = .049	0.00 (−0.01 to 0.01); *p* = .596
Model 4: (n = 2,445)	0.03 (–0.03 to 0.09); *p* = .307	0.04 (0.01–0.09); *p* = .065	0.07 (0.02–0.15); *p* = .023	0.03 (−0.01 to 0.09); *p* = .222	0.01 (−0.00 to 0.04); *p* = .432

a Model 1a shows the unadjusted results using the full imputed dataset; model 1b shows results after adjusting for confounders assessed in pregnancy; model 2 shows the unadjusted results using imputed data for those offspring who were sent the questionnaire at age 16 years; model 3 shows the unadjusted results using imputed data for mediators in those who had complete outcome data; model 4 shows the unadjusted results using only those with complete data on all variables in analysis.

b Adjusting for confounders assessed in pregnancy (child gender, housing tenure, marital status, maternal level of education, smoking in pregnancy, maternal family history of depression, and maternal psychiatric disorder before pregnancy).
