# Supplementary material for: Pathways to Suicide-Related Behavior in Offspring of Mothers With Depression: The Role of Offspring Psychopathology
Source: J Am Acad Child Adolesc Psychiatry. 2015 May;54(5):385–93. doi: 10.1016/j.jaac.2015.02.006 (PMC4411216; doi:10.1016/j.jaac.2015.02.006)
Supplement: Supplementary Data [file mmc1.docx]

**Supplement 1**

*Derivation of Latent Classes of Maternal Depression Symptoms*

Latent class growth analysis (LCGA) was used to identify qualitatively distinct patterns of depression symptoms in mothers over time from 18 weeks’ gestation to child age 11 years using the Edinburgh Postnatal Depression Scale (EPDS). In LCGA, each mother is given a probability of belonging to each class, and these probabilities are then used to assign each mother to her most likely class. Based on fit statistics, size of the latent classes, and parsimony, a 5-class model represented the best fit to the data. Approximately 5% of the sample was identified as belonging to a class with high, stable symptoms that were consistently above the clinical cut-off of 13 on the EPDS (chronic-severe class). Nearly 18% belonged to a class with subthreshold symptoms over time, with symptom levels that were consistently just below the clinical cut-off on the EPDS and decreased very slightly over time (subthreshold class). Just under 6% belonged to a class with increasing symptoms over time, with symptom levels that rose to the clinical cut-off by the last time point (increasing class). Around one-third of the sample (32%) belonged to a class with stable mild symptoms over time (mild class). Finally, the majority of the sample (40%) belonged to a class with very low levels of depression symptoms over time (minimal class).

In a previous study (Hammerton et al; unpublished manuscript), it was found that offspring of mothers from each of the depression classes were at increased risk for later suicide-related behavior compared to offspring of mothers with minimal symptoms. In addition, it was found that offspring of mothers with chronic-severe symptoms were at greatest risk; however, there was no difference in risk between offspring of mothers with mild, increasing, and subthreshold symptoms. Therefore, the 5 classes were collapsed to form a 3-level categorical variable: 0 = minimal; 1 = moderate (including mild, increasing, and subthreshold classes); 2 = chronic-severe. The patterns of association among the 3 classes of maternal depression symptoms and frequency of maternal suicide attempt, past psychiatric disorder, family history of depression, and demographics were consistent with the patterns expected (Table S1).

**Table S1**Pattern of Maternal Suicide Attempt, Psychiatric Disorder Before Pregnancy, Family History of Depression, Housing Tenure, Marital Status, Smoking in Pregnancy, and Level of Education by Classes of Maternal Depression Symptoms

| **Characteristic** | **Minimal** | **Moderate** | **Chronic-severe** |
| --- | --- | --- | --- |
| Maternal suicide attempt | 0.34 | 2.32 | 10.87 |
| Maternal psychiatric disorder before pregnancy | 4.59 | 13.87 | 37.63 |
| Maternal family history of depression (both parents) | 1.23 | 2.52 | 5.97 |
| Housing tenure (rented) | 16.05 | 23.98 | 37.08 |
| Marital status (single) | 16.54 | 23.97 | 32.37 |
| Smoked in pregnancy | 16.86 | 25.31 | 35.92 |
| Maternal education (<O-level) | 24.27 | 27.25 | 36.76 |

*Note:* All data are given as percentages. Imputed N = 10,559.

**Table S2** Mean Depression Symptoms (With 95% CIs) at Each Assessment for Mothers in the Chronic-Severe Class, Moderate Class, and Minimal Class

| **Child Age at EPDS Assessment** | **Mean (95% CI)** | | |
| --- | --- | --- | --- |
|  | **Chronic-Severe class (5.2%)** | **Moderate Class (55.2%)** | **Minimal Class (39.6%)** |
| 18 wk gestation | 14.3 (13.9–14.7) | 8.4 (8.3–8.5) | 3.5 (3.4–3.6) |
| 32 wk gestation | 15.5 (15.1–15.9) | 8.7 (8.6–8.8) | 3.3 (3.2–3.4) |
| 8 wk | 14.8 (14.4–15.2) | 7.5 (7.4–7.6) | 2.6 (2.5–2.7) |
| 8 mo | 14.7 (14.3–15.1) | 6.9 (6.8–7.0) | 2.0 (2.0–2.1) |
| 21 mo | 15.5 (15.2–15.9) | 7.3 (7.2–7.4) | 2.3 (2.2–2.4) |
| 33 mo | 15.7 (15.3–16.1) | 8.0 (7.9–8.2) | 2.6 (2.6–2.7) |
| 61 months | 15.4 (15.0–15.9) | 7.7 (7.6–7.8) | 2.6 (2.5–2.6) |
| 73 mo | 15.6 (15.2–16.1) | 8.1 (7.9–8.2) | 2.8 (2.7–2.9) |
| 97 mo | 15.6 (15.0–16.1) | 7.7 (7.6–7.8) | 2.7 (2.6–2.8) |
| 134 mo | 15.2 (14.7–15.8) | 7.4 (7.2–7.5) | 2.6 (2.5–2.8) |

*Note:* N = 10,559; clinical cut-off on Edinburgh Postnatal Depression Scale (EPDS) = 13.

**Supplement 2**

*Missing Data Imputation Using Multivariate Imputation by Chained Equations (MICE)*

Missing data were imputed with a fully conditional specification using the MICE^1^ algorithm in STATA 13. Previous studies have recommended using multiple imputation to deal with potential bias arising from missing data, especially when data are thought to be missing at random (MAR; i.e., given the observed data, the missingness mechanism does not depend on the unobserved data^2^). As the Avon Longitudinal Study of Parents and Children (ALSPAC) sample has substantial information on socio-demographic variables that predict missingness, missing information can be assumed to be dependent on observed data. Those participants with missing information on outcome and mediators differed from the starting sample on a number of demographic characteristics. Mothers were younger, and they were also more likely to smoke during pregnancy, live in rented accommodation and crowded housing, be single, have a lower socioeconomic status, lower education, and increased parity. Offspring were less likely to be female. Therefore, these variables were included in the imputation model to make the assumption of MAR as plausible as possible. The imputation model also included other measures that have been found to be closely associated with offspring suicide-related behavior and psychopathology (such as multiple measures of offspring suicidal ideation and self-harm at other ages, depression, anxiety, disruptive behavior, and ADHD diagnoses and symptoms at multiple time points and using multiple informants and measures of substance abuse; full list available on request) and all other variables included in the analyses.^1^

Using binary and multinomial logistic and linear regression models as appropriate, 80 imputed datasets were derived, each with 10 cycles of regression switching. Predictive mean matching was used when continuous variables were not normally distributed. Where subgroups were analyzed, data were imputed separately by group (for example, by presence of suicidal ideation at age 11 years). All analyses were then run on imputed datasets by combining estimates using Rubin’s rules.^1^ It has been recommended that the number of imputed datasets exceeds 100*the maximum fraction of missing information (FMI) value. FMI values were found to be no larger than 0.7; therefore, imputing 80 datasets is adequate.^1^

All variables with missing data used in analyses were imputed up to the maximum sample size of 10,559. A number of sensitivity checks were performed by repeating analyses using alternative approaches to dealing with missing data. First, analyses were rerun on those with complete data (n = 2,445); second, data were imputed only up to the sample for which complete outcome data were available (n = 4,588), and finally, data were imputed up to the sample of offspring who were sent the questionnaire measure at age 16 years (n = 8,475). Table S3 shows demographics for those with complete data (n = 2,445) and each of the imputed samples (n = 4,588; n = 8,475; N = 10,559) in comparison to the original ALSPAC cohort who met inclusion criteria for this study (n = 13,617). As shown in Table S3, the imputation procedure has corrected for biases present from selective attrition, with the imputed sample being more representative of the original ALSPAC cohort than the complete case sample.

**Supplemental References**

1. White IR, Royston P, Wood AM. Multiple imputation using chained equations: issues and guidance for practice. Stat Med. 2011;30:377-399.

2. Moodie EEM, Delaney JAC, Lefebvre G, Platt RW. Missing confounding data in marginal structural models: a comparison of inverse probability weighting and multiple imputation. Int J Biostat. 2008;4.

**Figure S1** Flow chart of retention in the Avon Longitudinal Study of Parents and Children (ALSPAC) sample. Note: EPDS = Edinburgh Postnatal Depression Scale.


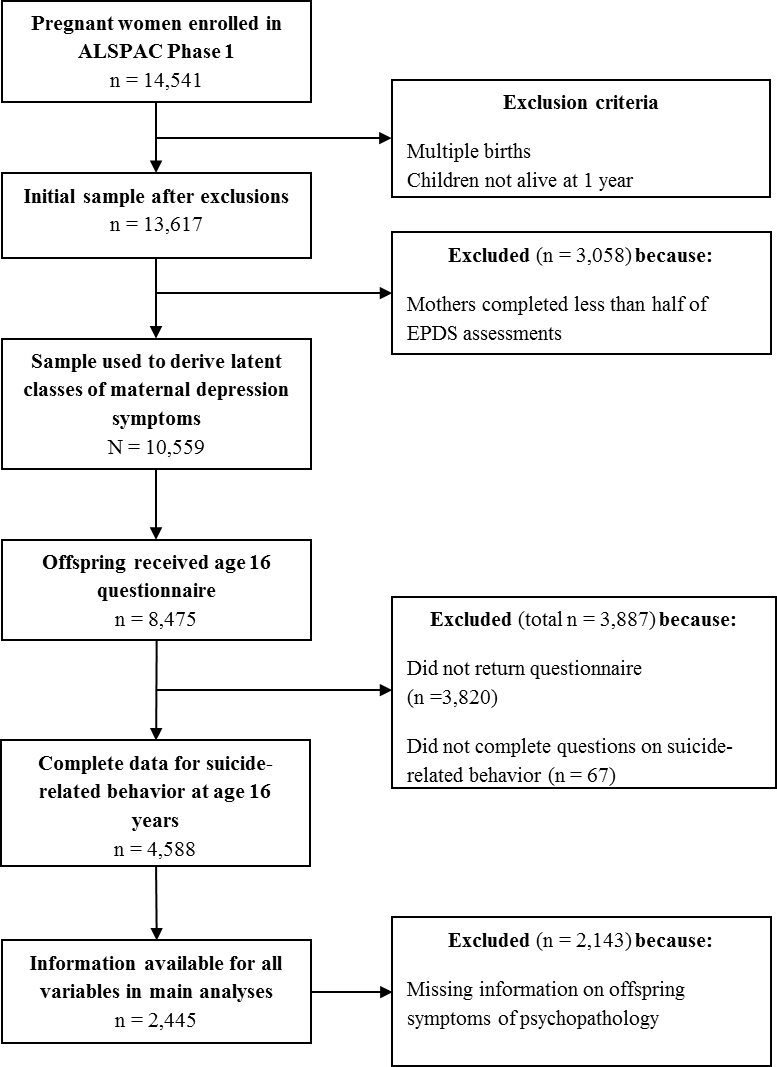


**Table S3** Demographics of the Complete Case Sample, 3 Imputed Samples, and the Original Cohort That Met Inclusion Criteria

| **Sample Demographics Assessed During Pregnancy^a^** | **Complete Cases^b^**  **(n = 2,445)** | **Imputed Sample^c^**  **(n = 4,588)** | **Imputed Sample^d^**  **(n = 8,475)** | **Imputed Sample^e^**  **(N = 10,559)** | **Initial Cohort^f^**  **(n ≤ 13,617)** |
| --- | --- | --- | --- | --- | --- |
| Female offspring (%) | 56.9 | 58.5 | 50.5 | 48.4 | 48.4 |
| Smoked in pregnancy (%) | 12.4 | 16.6 | 20.7 | 22.5 | 25.8 |
| Housing tenure (% rented) | 11.0 | 14.5 | 19.1 | 21.5 | 26.7 |
| Marital status (% single) | 15.0 | 17.0 | 19.8 | 21.5 | 23.5 |
| Maternal education (% <O-level) | 14.7 | 18.4 | 24.2 | 26.6 | 26.7 |
| Maternal depression (mean EPDS score at 32 wk gestation) | 6.07 | 6.36 | 6.77 | 6.90 | 7.05 |

*Note:* EPDS = Edinburgh Postnatal Depression Scale.

^a^Additional missing data on demographics: smoked in pregnancy missing for 792 of 13,617; housing tenure missing for 914 of 13,617; marital status missing for 858 of 13,617; maternal education missing for 1,515 of 13,617; maternal EPDS missing for 1,895 of 13,617.

^b^Sample with complete data on maternal depression classes, offspring suicide-related behavior, and symptoms of psychopathology.

^c^Sample with imputed data for mediators and confounders in those who had complete outcome data.

^d^Sample with imputed data for mediators, confounders, and outcome in those offspring who were sent the questionnaire at age 16 years.

^e^Sample with imputed data for mediators, confounders, and outcome in those who that had complete exposure data (for those who have information on latent classes of maternal depression symptoms; main sample used throughout Results section of main article).

^f^Original Avon Longitudinal Study of Parents and Children (ALSPAC) cohort that met inclusion criteria for this study.

**Table S4** Means, Standard Deviations, and Correlations Between Offspring Symptoms of Psychopathology

| **Symptoms** | **1** | **2** | **3** | **4** | **5** |
| --- | --- | --- | --- | --- | --- |
| 1. MDD symptoms | — |  |  |  |  |
| 2. GAD symptoms | 0.52*** | — |  |  |  |
| 3. DBD symptoms | 0.21*** | 0.14*** | — |  |  |
| 4. ADHD symptoms | 0.12*** | 0.09*** | 0.59*** | — |  |
| 5. Alcohol abuse symptoms | 0.22*** | 0.11*** | 0.29*** | 0.21*** | — |
| Mean (SD) | 3.06 (6.10) | 2.87 (5.63) | 1.54 (5.52) | 2.59 (6.77) | 3.22 (11.22) |

*Note:* Imputed N = 10,559. ADHD = attention-deficit/hyperactivity disorder; DBD = disruptive behavior disorder; GAD = generalized anxiety disorder; MDD = major depressive disorder.

****p* < .001.

**Table S5** Univariate Linear and Logistic Regression Analyses Between Each Class of Maternal Depression Symptoms in Comparison to Minimal Class (Reference Group) and Offspring Symptoms of Psychopathology at Age 15 Years

| **Maternal Depression Class (Exposure)** | **β (95% CI)** | | | | |  |  |  |
| --- | --- | --- | --- | --- | --- | --- | --- | --- |
|  | **MDD Symptoms** | **GAD Symptoms** | **DBD Symptoms** | **ADHD Symptoms** | **Alcohol Abuse Symptoms** |  |  |  |
| Minimal | Reference group | | | | |  |  |  |
| Moderate | 0.17 (0.11–0.22)*** | 0.18 (0.13–0.23)*** | 0.29 (0.24–0.34)*** | 0.31 (0.26–0.36)*** | 0.10 (0.04–0.16)*** |  |  |  |
| Chronic-severe | 0.38 (0.25–0.51)*** | 0.41 (0.27–0.56)*** | 0.80 (0.66–0.94)*** | 0.75 (0.62–0.87)*** | 0.29 (0.15–0.43)*** |  |  |  |

*Note:* Imputed N = 10,559. ADHD = attention-deficit/hyperactivity disorder; DBD = disruptive behavior disorder; GAD = generalized anxiety disorder; MDD = major depressive disorder.

**** p* ≤ 0.001.

**Table S6** Indirect Effect of Maternal Moderate Depression Symptoms (With Minimal Class as the Reference Group) on Offspring Suicidal Ideation Through Offspring Symptoms of Major Depressive Disorder (MDD), Generalized Anxiety Disorder (GAD), Disruptive Behavior Disorder (DBD), Attention-Deficit/Hyperactivity Disorder (ADHD), and Alcohol Abuse

| **Model ^a^** | **Indirect Effects via Offspring Symptoms (Probit Coefficient [95% CI])** | | | | |
| --- | --- | --- | --- | --- | --- |
|  | **MDD** | **GAD** | **DBD** | **ADHD** | **Alcohol Abuse** |
| Model 1a: using full imputed data; unadjusted (N = 10,559) | 0.05 (0.03–0.06); *p* < .001 | 0.03 (0.02–0.04); *p* < .001 | 0.04 (0.02–0.06); *p* < .001 | −0.01 (−0.03 to 0.01); *p* = .284 | 0.01 (0.00–0.01); *p* = .030 |
| Model 1b: adjusted for confounders (N = 10,559) ^b^ | 0.04 (0.02–0.05); *p* < .001 | 0.02 (0.01–0.03); *p* < .001 | 0.03 (0.02–0.04); *p* < .001 | −0.00 (−0.02 to 0.01); *p* = .741 | 0.00 (−0.00 to 0.01); *p* = .195 |
| Model 2: as model 1a, imputing those who were sent questionnaire (n = 8,475) | 0.05 (0.03–0.06); *p* < .001 | 0.03 (0.01–0.04); *p* < .001 | 0.04 (0.02–0.05); *p* < .001 | −0.01 (−0.03 to 0.01); *p* = .200 | 0.01 (0.00, 0.01); *p* = .041 |
| Model 3: as model 1a, imputing those with complete outcome data (n = 4,588) | 0.04 (0.02–0.06); *p* < .001 | 0.02 (0.01–0.03); *p* = .001 | 0.03 (0.01–0.05); *p* < .001 | −0.01 (−0.03–0.01); *p* = .222 | 0.00 (−0.00 to 0.01); *p* = .131 |
| Model 4: as model 1a, complete cases (n = 2,445) | 0.03 (0.01–0.05); *p* = .004 | 0.02 (0.01–0.03); *p* = .015 | 0.03 (0.02–0.06); *p* < .001 | −0.02 (−0.05 to −0.01); *p* = .017 | 0.00 (−0.00 to 0.01); *p* = .777 |

*Note:* ^a^ Model 1a shows the unadjusted results using the full imputed dataset; model 1b shows results after adjusting for confounders assessed in pregnancy; model 2 shows the unadjusted results using imputed data for those offspring who were sent the questionnaire at age 16 years; model 3 shows the unadjusted results using imputed data for mediators in those who had complete outcome data; model 4 shows the unadjusted results using only those with complete data on all variables in analysis.

^b^ Adjusting for confounders assessed in pregnancy (child gender, housing tenure, marital status, maternal level of education, smoking in pregnancy, maternal family history of depression, and maternal psychiatric disorder before pregnancy.
